# Current Concepts of Pediatric Acute Kidney Injury—Are We Ready to Translate Them into Everyday Practice?

**DOI:** 10.3390/jcm10143113

**Published:** 2021-07-15

**Authors:** Kinga Musiał

**Affiliations:** Department of Pediatric Nephrology, Wrocław Medical University, Borowska 213, 50-556 Wrocław, Poland; kinga.musial@umed.wroc.pl

**Keywords:** furosemide stress test, hyperfiltration, [IGFBP-7] × [TIMP-2], NGAL, renal angina index, renal functional reserve, tubular damage, tubuloglomerular feedback

## Abstract

Pediatric acute kidney injury (AKI) is a major cause of morbidity and mortality in children undergoing interventional procedures. The review summarizes current classifications of AKI and acute kidney disease (AKD), as well as systematizes the knowledge on pathophysiology of kidney injury, with a special focus on renal functional reserve and tubuloglomerular feedback. The aim of this review is also to show the state-of-the-art in methods assessing risk and prognosis by discussing the potential role of risk stratification strategies, taking into account both glomerular function and clinical settings conditioned by fluid overload, urine output, or drug nephrotoxicity. The last task is to suggest careful assessment of eGFR as a surrogate marker of renal functional reserve and implementation of point-of-care testing, available in the case of biomarkers like NGAL and [IGFBP-7] × [TIMP-2] product, into everyday practice in patients at risk of AKI due to planned invasive procedures or treatment.

## 1. Introduction

Pediatric acute kidney injury (AKI) is a serious clinical condition, associated with increased morbidity and mortality, as reported in large observational studies like AWARE (Assessment of Worldwide Acute Kidney Injury, Renal Angina, and Epidemiology) or AWAKEN (Assessment of Worldwide Acute Kidney Injury Epidemiology in Neonates) [1,2,3]. Yet, defining an efficient and reliable tool for kidney injury assessment in children is still a challenge. Despite longitudinal efforts and a wide range of methods tested in adults, the results seem difficult to be transferred directly into the pediatric background, where the results and their interpretation are age-dependent. While methods of evaluating renal function in stable milieu are established, the real task is how to predict the kidney ability to preserve function despite injury under stress conditions. Thus, we should search for static and dynamic markers of kidney function, as well as parameters of functional flexibility, predicting recovery after injury.

The history of progress in diagnosing kidney injury and predicting prognosis screens through various classifications, identification of risk factors, discovery and increasing role of damage biomarkers, evaluation of renal functional reserve and usage of stress tests, and finally search for predictors of progression from AKI to CKD or of recovery. The major task for the future is how to translate the adult experience into the pediatric clinical setting in order to prevent kidney injury or provide recovery from it.

## 2. Classical AKI Definitions

Defining acute kidney injury was a milestone towards understanding the nature of renal damage. The goal behind subsequently developed criteria (RIFLE, pRIFLE, AKIN) was to find the most accurate tool for early diagnosis and efficient treatment of AKI [4,5,6]. Finally, the KDIGO AKI definition was developed as a unified classification for children and adults and is now recommended for pediatric AKI [7,8,9] (Table 1).

However, all these classifications still depend mainly on serum creatinine, an imperfect kidney function evaluation tool. Indeed, creatinine clearance may reflect eGFR in steady conditions, but stress modifies its value owing to creatinine excretion by proximal tubules. Moreover, creatinine concentration is biased with multiple modifiers (muscle mass, metabolism, hydration status, use of diuretics) and delayed toward actual time of injury. Thus, further research has concentrated on finding markers that would precede creatinine rising. The whole domain of injury biomarkers has evolved from a new understanding of AKI, according to which functional loss and damage may follow each other or exist concurrently [10].

## 3. Expanded AKI Definition

**Figure 1:**
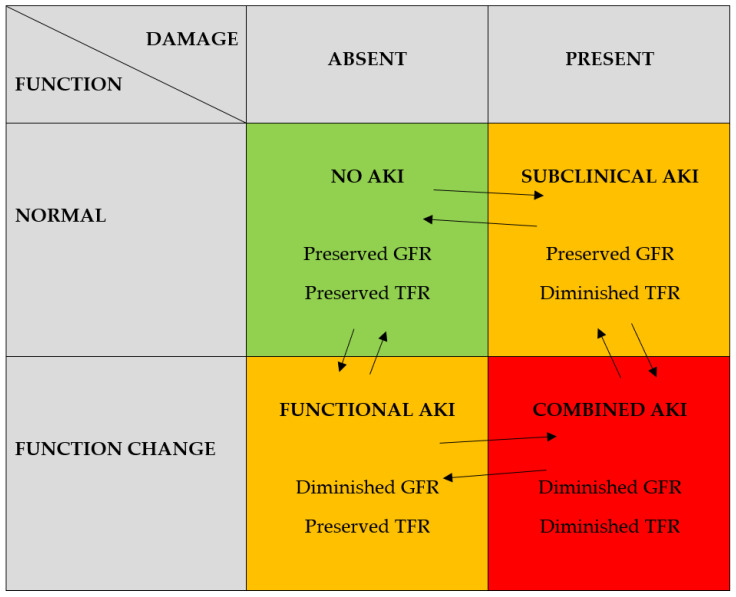
Expanded acute kidney injury (AKI) criteria combining function and damage biomarkers, glomerular functional reserve (GFR), and tubular functional reserve (TFR) (according to [10], modified).

Recommendations given by the 10th Acute Dialysis Quality Initiative (ADQI) have combined functional and damage markers and created four categories in the AKI field [10]. The classification distinguishes between loss of glomerular function and presence of tubular damage, showing that they may appear separately, subsequently, or concomitantly [10]. This new classification underlines the idea of transition between categories and assessment of the kidney status over time. The term of “functional AKI”, where glomerular filtration rate is decreased, but markers of damage are absent, covers all cases of volume-dependent, time-sensitive, and potentially reversible alterations in kidney function preceding damage. Such transient elevation of serum creatinine could be observed in the early phases of “pre-renal azotemia” resulting from dehydration or “post-renal AKI” with urinary tract obstruction without signs of damage. The idea of “subclinical AKI” is new, stressing the fact that damage may precede functional loss, like in the case of drugs with nephrotoxic potential (Figure 1). Therefore, damage markers may become promising early predictors of incipient AKI. The “combined AKI” category unified the two former conditions, whereas “no AKI” was complementary to normal renal function without signs of damage (Figure 1). Moreover, the authors proposed a hypothesis that all options may turn into one another, thus defining various types of AKI-associated injury as potentially reversible processes [10].

## 4. Markers of Injury

The spectrum of AKI biomarkers in the pediatric population has been reviewed extensively elsewhere [11,12,13,14]. However, their implementation is restricted to small groups of patients and the results, rather of scientific than diagnostic value, could not modify the strategy of improvement for pediatric AKI [15]. Thus, despite the robustness of markers tested in the AKI conditions, only a few proved their usefulness in the prediction of AKI or prediction of recovery and found their place in everyday practice. Out of a wide range of candidates, only NGAL and [IGFBP7] × [TIMP-2] product were put into the form of point-of-care testing, thus serving as diagnostic tools.

### 4.1. Neutrophil Gelatinase-Associated Lipocalin (NGAL)

NGAL is a low molecular weight protein (25 kDa), filtered freely by glomeruli and reabsorbed by the proximal tubules. The distal tubular epithelial cells are the main source of NGAL, but its urinary increase is a sign of proximal tubule injury, especially due to ischemia. AKI triggers the release of a monomeric form into the urine. Serum, plasma, and urine NGAL are useful in predicting AKI, although recent meta-analysis has confirmed the superiority of urine NGAL over serum/plasma measurements (area under the curve (AUC) = 0.92 for urine NGAL, AUC = 0.87 for serum NGAL, and AUC = 0.84 for plasma NGAL) in patients with sepsis [16]. Urine NGAL also performed well in the pediatric population. It could predict 30-day and 3-month mortality in children with AKI [17], and its concentration could distinguish between patients at risk of developing AKI as a consequence of nephrotoxicity [18]. Most recently, uNGAL has shown its usefulness, together with CT-scan, in the assessment of AKI in patients suffering from SARS-CoV 2 infection [19].

### 4.2. Insulin-Like Growth Factor-Binding Protein (IGFBP)-7

IGFBP-7 is another low molecular weight (30 kDa) protein freely filtered by glomeruli and reabsorbed by proximal tubules. Renal injury results in its increased tubular expression and tubular cell cycle arrest in the G1 phase. The consequence is decreased energy consumption in the course of a self-protective mechanism. Increased IGFBP-7 urinary concentrations were noticed in children and neonates from intensive care units. They predicted renal outcome and distinguished between patients with and without AKI, or between those with early recovery from AKI and late or non-recovery [20,21].

### 4.3. Tissue Inhibitor of Metalloproteinase (TIMP)-2

TIMP-2 (21 kDa), similarly to IGFBP-7, is another marker of cell cycle arrest. It proved its superiority over damage markers, like urinary NGAL, KIM-1, or L-FABP, in AKI prediction among critically ill patients. The assessment of [IGFBP-7] × [TIMP-2] product gave even better results in the prediction of progression to mild/severe AKI and of 30-day and 3-month mortality due to AKI [20,21,22]. Moreover, similarly to NGAL, [IGFBP-7] × [TIMP-2] is now available in the form of a point-of-care test, Nephrocheck. Nalesso et al. [23] have analyzed the usefulness of Nephrocheck based on the literature analysis. The authors concluded that the use of this test should be considered in the case of any large surgery or when invasive procedure is planned. The major restriction is the fact that the predictive value of [IGFBP-7] × [TIMP-2] product increases with the severity of patient condition. In practice, the marker distinguishes quite precisely between those who develop severe AKI and no AKI, but copes rather poorly with the prediction of mild forms of AKI [23].

The above-mentioned examples clearly show that the biomarker universum is still under construction. Additionally, the ongoing discussion shows large discrepancies among scientists and clinicians regarding their accuracy and predictive value [24,25,26]. Yet, their diagnostic value is restricted to the short period between pre-AKI and AKI time points.

## 5. From AKI into Acute Kidney Disease (AKD)

Adding markers of injury to the current understanding of AKI has expanded diagnostic possibilities [10]. However, AKI milieu according to KDIGO criteria is restricted to one week from the set point of injury, concentrating on the dynamics and progression of damage. The current concept of kidney dysfunction takes into account both pre-injury and post-injury conditions, putting stress on factors predicting damage and assessing a chance for recovery. The latter seems of paramount prognostic value, distinguishing between potentially reversible acute injury and irreversible chronic kidney disease (CKD).

The consensus report of the Acute Disease Quality Initiative (ADQI) has aimed at describing conditions of ongoing pathophysiological process in the kidney after AKI [27]. According to ADQI, acute kidney disease (AKD) encompasses all episodes of kidney injury persisting for more than 7 days, but less than 90 days [27].

The current KDIGO definition of AKD takes into account the exclusive or concurrent persistence of serum creatinine rise/eGFR decrease/damage marker presence [8] (Table 1). ADQI consensus has divided AKD into stages, congruent with AKI stages 1–3 (Table 2). Additionally, “subacute AKD”, called stage zero, was added and subdivided into three categories, depending on the unchanged/increased serum creatinine or absence/presence of damage markers (Table 2).

As the AKI definition requires strict time limits that do not fit every patient with kidney damage, AKD seems to have a chance to classify those who did not fulfill the criteria of AKI, but demonstrate persistent features of renal injury. Moreover, an extended observation period is essential in establishing the future direction of changes—to recovery or toward irreversible damage.

Indeed, ADQI has proposed several hypothetical trajectories of AKD sequelae from day 0 (injury) to day 28. Their range was from rapid reversal of normal kidney function within 48 h to systematic progression from subacute AKI towards AKD stage 3 within 28 days [27]. Of note, those who developed AKI stage 3 within the first 48 h could partially improve within 7 days to AKI stage 2, then to AKD stage 2, and finally to stage 1. However full recovery was not possible [27].

## 6. AKI–AKD–CKD Continuum

The above-mentioned hypothetical models aimed to show the idea of the continuum between AKI, AKD, and CKD, where AKD is a link between AKI and CKD [27]. However, AKD in this concept should be understood as an AKI sequel, with an ongoing recovery or damage and possibility of either favorable or adverse consequences. Treating AKD like a CKD prequel may trigger an unexpected bias, because, among all definitions of kidney injury, only CKD bears the burden of irreversibility.

### AKI in the Presence of Pre-Existing CKD

All AKI definitions concentrate on the actual kidney dysfunction without considering potential previous episodes of injury. Such conjecture is justified in the situation when the focus is on the proportional change in serum creatinine or estimated glomerular filtration rate, rather than on their absolute post-exposure values. The latter may be biased with different threshold values when current injury develops on the basis of chronic kidney disease. Moreover, the ability of the kidney to use its functional reserve may maintain apparently normal eGFR values despite ongoing kidney function decline. Silent loss of nephrons during the AKI–AKD–CKD continuum may be difficult to perceive, but current knowledge provides tools to diagnose this process.

## 7. Renal Functional Reserve

The term renal functional reserve (RFR) was established in the 1980s to describe the difference between the baseline eGFR value and its increase after stimulation by protein intake [28]. The concept of discrepancy between kidney function in stable versus stress conditions originates from the observation that baseline kidney capacity is at approximately 75% of its maximal filtration [28,29]. Such reserve secures quick adaptation to physiological demands, like protein intake or excessive/insufficient fluid supplementation. A similar ability allows to compensate transient decrease in functioning renal mass during pathological conditions. The value of basal eGFR and the extent to which it can increase in stress conditions both depend on the intact renal mass. In consequence, return to the baseline eGFR value after a temporary decline in the course of a single episode of AKI can be achieved, although it is reached at the cost of reduced RFR [30]. Repeat AKI episodes may lead to continuous mobilization of RFR to maintain eGFR. Such worsening of kidney function would pass unnoticed until RFR becomes exhausted with subsequent injuries. It is assumed that the loss of functioning nephrons may remain undiagnosed, owing to unchanged eGFR values, as long as the intact renal mass is reduced by no more than 50%. Beyond this threshold, the rate of kidney damage may grow exponentially [29].

### The Mechanism of eGFR Increase

The mechanism by which eGFR increases is triggered depending on the presence/absence of underlying renal injury. Physiologically, stress conditions evoke additional recruitment of intact nephron units and subsequent increased filtration, as observed during pregnancy, in patients with solitary kidney or in those who became living related donors. This apparently normal eGFR is maintained at the cost of RFR stimulation. Under pathological conditions, the reduced mass of functioning nephrons can only respond by augmented filtration of a single nephron, resulting in hyperfiltration [29]. Such a scenario accompanies obesity, diabetic nephropathy, or primary glomerulopathies. Yet, it is also based on persistent stimulation of RFR. In consequence, decompensation of regulatory mechanisms may follow.

Indeed, large observational studies concluded that, even in health, the presence of hyperfiltration signifies further eGFR decline and risk of CKD development [31,32]. The Japanese study revealed that basic eGFR values in people with hyperfiltration were significantly higher (although within normal range) than in those who did not develop hyperfiltration [31]. Moreover, those increased values persisted for 3.3 ± 1.9 years before they reached the threshold of hyperfiltration. Then, the eGFR decline followed, and it was accelerated in patients with hyperfiltration when compared with those without it [31]. The Korean experience proved that hyperfiltration was connected with the increased risk of incident proteinuria and 30% eGFR decline [32]. The authors concluded that hyperfiltration may be treated as an indicator of increased risk of developing CKD in an apparently healthy population. The study held in Singapore has strengthened the above conclusions by showing that the risk of renal decline was even higher in patients with hyperfiltration caused by diabetes [33]. Summing up, the expense of hyperfiltration is costly even in the healthy population, although those with comorbidities pay more.

These observations urge to describe the phenomenon of hyperfiltration. The hypothetical process has been analyzed in detail in the case of oral protein load or amino acid infusion [30,34]. The major force triggering hyperfiltration in this case is tubuloglomerular feedback.

## 8. Tubuloglomerular Feedback

Tubuloglomerular feedback (TGF) is an autoregulatory mechanism to control glomerular filtration at a single nephron level. The major goal for TGF is to maintain glomerular capillary pressure within a safe range. The control of vascular tone within the afferent arteriole is adjusted to water and salt delivery to the distal tubule sensing site—macula densa. When distal tubular delivery of NaCl aggravates, macula densa triggers vasoconstriction of the above mentioned artery, glomerular capillary pressure decreases, and eGFR value declines.

Diabetes is the most spectacular example of interactions between tubules and glomeruli, with subsequent hyperfiltration and diabetic kidney disease [35]. When the increased content of filtered glucose reaches proximal tubules, sodium-glucose cotransporters 2 and 1 (SGLT2 and SGLT1) are responsible for enhanced reabsorption of glucose, together with sodium, chloride, and fluid [35]. This phenomenon is aggravated by tubular hypertrophy, resulting from hyperglycemia, and subsequent increased expression of high-capacity transporter SGLT2 in the early proximal tubule. Decreased delivery of Na, Cl, and fluid to macula densa triggers tubuloglomerular feedback with hyperfiltration. Additionally, reduced fluid content, reaching distal tubule, decreases hydrostatic back pressure in the Bowman’s capsule, thus enhancing glomerular filtration and physical stress on the filtration barrier [35].

Oral protein intake or amino acid infusion result in the increased filtered load of amino acids and their aggravated reabsorption, together with NaCl, by proximal tubules [30]. In consequence, diminished delivery of NaCl to distal tubules senses macula densa to inhibit the activity of tubuloglomerular feedback and evoke hyperfiltration through vasodilatation of the afferent artery. Preglomerular vessel dilatation provokes increase in renal plasma flow (RPF), without changing filtration fraction. Among factors responsible for this phenomenon, paracrine and endocrine stimuli seem of paramount importance. One of the major regulators in TGF is nitric oxide (NO).

Amino acid infusion increases the intrarenal synthesis of nitric oxide (NO). Experimental data have proven the direct impact of neuronal nitric oxide synthase β (NOS1 β) from macula densa on TGF and subsequent change in eGFR [36]. Wild-type mice on a 4-week high-protein diet demonstrated kidney hypertrophy, glomerular hyperfiltration, increased blood flow, decreased renal vascular resistance, as well as upregulated expression and activity of macula densa NOS1 β, in comparison with mice fed a low-protein diet. Meanwhile, the TGF response was blunted both in vivo (measurement by micropuncture) and in vitro (measurement of diameters of the afferent artery), but to a greater extent in mice on the high-protein diet. Macula densa NOS1 β knockout mice demonstrated attenuation of all these reactions despite high-protein diet intake [36].

Although TGF is most efficient in response to short-term stimuli, the above mentioned results suggest that chronic TGF stimulation may establish a new set point of distal tubular flow. In practice, TGF becomes less sensitive to increased NaCl load and single nephron eGFR remains increased. Such a concept would explain the tight connections between tubules and glomeruli, not only in acute conditions, but also as a consequence of chronic kidney injury, leading, e.g., to diabetic kidney disease.

## 9. Tubular Function Testing

Renal functional reserve provides the information on maximal capacity of glomerular filtration in stress conditions. The major challenge in everyday practice is the fact that no routine test for clinical assessment of RFR exists. Once TGF was revealed, it became clear that eGFR value is tightly connected with proximal tubule function. Having said that, is it worth testing tubular function as an equivalent of renal capacity to adapt to unfavorable conditions? The concept of the tubular stress test is parallel to the glomerular stress test after protein/amino acid load—tubular secretion of creatinine is assessed after protein meal and confronted with the baseline secretion value [37]. Although available in clinical practice, this test still requires standardization.

### Furosemide Stress Test

Contrary to the tubular stress test, the furosemide stress test is used routinely to assess tubular damage in patients with AKI. Recent evidence shows that its result can be interpreted as a predictive tool in assessing the risk of progression into AKI stage 3 and need for renal replacement therapy. The concept is based on loop diuretic pharmacodynamics. Furosemide is secreted by proximal tubules and acts at the intraluminal side of the ascending limb of the loop of Henle, where it inhibits the Na K Cl2 cotransporter and induces natriuresis [37]. In practice, 1–1.5 mg/kg b.w. of Furosemide is given and urine output is assessed after 2 h [37]. Volume exceeding 200 mL is a predictor of good prognosis.

The above-mentioned tests should open the discussion about introducing tubular functional reserve into the future panel of renal injury/recovery markers. Glomerular functional reserve and tubular functional reserve, analyzed together with damage markers, may complete the picture of kidney dysfunction (Figure 1).

## 10. Risk Stratification Strategy

Combined glomerular and tubular function assessment in static and dynamic conditions does not provide a complete evaluation of the patient’s current status or prognosis. Clinical perspective is essential and cannot be neglected. The complex analysis of biomarker/stress test/functional reserve interrelations, together with the clinical context, has evolved into various evaluating systems able to stratify the risk of AKI development/progression to AKI stage 3/progression to CKD/recovery. The above-mentioned complex analysis is best illustrated by two systems of risk evaluation: renal angina index (RAI) and fluid overload kidney injury score (FOKIS).

### 10.1. Renal Angina Index (RAI)

RAI was created in order to recognize patients at risk of developing AKI within 72 h [38]. The classification categorizes patients according to two major features: risk and injury. Within the risk category, scoring encompasses moderate (one point), high (three points), and very high (five points) risk. Moderate risk is connected with admission to pediatric intensive care unit, high risk encompasses patients after stem cell transplantation, whereas very high risk patients are on ventilation. The category of injury identifies changes in creatinine clearance and fluid overload [38]. One point is dedicated to patients with unchanged kidney function and moderate (<5%) fluid overload (FO). The two-point evaluation is given to those whose eGFR has decreased by no more than 25%, while FO has exceeded 5%. Four points describe patients with 25–50% eGFR decrease and >5% FO. Finally, eight points means >50% eGFR decrease and >5% FO. The risk score is multiplied by the injury score, thus the range of points is from 1 to 40. Anyone obtaining more than eight points in the final assessment will most probably develop AKI within 72 h. The values below eight have a strong negative predictive value toward developing AKI in the next 3 days [38].

### 10.2. Fluid Overload Kidney Injury Score (FOKIS)

Fluid overload seriously worsens the prognosis in AKI. Pathologic accumulation of interstitial fluid in the kidney may additionally impair perfusion by the obstruction of capillary blood flow. Calculation of FO is based on multiple equations taking into account fluid balance or patient weight [39]. It is assumed that 10% FO is a threshold value for intervention.

FOKIS is a newly developed four-dimentional score putting together different categories: urine output (UOP), fluid overload (FO), serum creatinine, and nephrotoxin use [39]. Both UOP and changes in kidney function (eGFR decrease) are classified according to the pRIFLE categories and gain points from 0 to 3. The FO criteria range from <15% (1 point) to >35% (5 points). The category of nephrotoxins scores a patient treated with less than three nephrotoxic drugs (0 points), with three nephrotoxic medications (1 point), and allows to give another point (+1 point) for every additional nephrotoxic agent [39].

## 11. How to Prevent Kidney Injury in Children?

The above-mentioned tools stratify the risk of developing AKI based on clinical data and glomerular function. Yet, none of the classifications dedicated to children encompasses the use of biomarkers.

Relying on the values of estimated GFR while assessing kidney function in children may give discrepant results. AKI provides dynamic conditions and highly changeable concentrations of serum creatinine, delayed in their dynamics toward the time of injury. Age-dependent variability of its values increases potential bias. Therefore, an efficient future strategy facing current demands should be implemented based on available tools.

The first attempt should be to properly evaluate renal function and functional reserve, taking into account the fact that these two strongly depend on one another. A patient with hyperfiltration has a decreased renal functional reserve, and thus is potentially at risk of developing AKI in unfavorable conditions. Thus, increased eGFR values cannot diminish the vigilance, whereas “normal” records should urge the search for past injuries and factors forcing mobilization of renal functional reserve.

One of the examples is the recently published data on renal function and AKI incidence in children undergoing hematopoietic stem cell transplantation (HSCT), who are classified by RAI as those at high risk of developing AKI. Most of the pediatric patients presented with hyperfiltration even before the procedure and its presence was correlated with previous chemotherapy [40]. Of note, the percentage of children with increased eGFR values was the highest among those who have undergone HSCT for oncological reasons. Moreover, urinary concentrations of tubular damage markers in these patients were significantly higher than in the age-matched healthy controls, even before HSCT, and rose after the procedure [41]. These observations suggested that children undergoing HSCT most probably approach this procedure with already diminished renal functional reserve. Such suggestions should urge to establish the next goal in AKI prevention among children. This task should be to implement the already available point-of-care tests, like urinary NGAL or [IGFBP-7] × [TIMP-2] product, in the risk groups prepared for planned interventions or procedures like stem cell transplantation or surgery.

The next move should be toward expanding the diagnostic abilities by forming “AKI panels”, allowing sequential assessment of various markers adjusted to the time after injury. Recent data show that combining serum creatinine, cystatin C, and urinary NGAL identifies better those children undergoing HSCT who are at risk of adverse outcomes [42]. This strategy would require establishment of reference ranges of damage markers for the pediatric population. Fortunately, first attempts have already been made, giving a chance for reliable results in the assessment of urinary NGAL, KIM-1, or L-FABP [43].

## 12. Conclusions

Current concepts of the nature of AKI show the complexity of interrelations between glomerular and tubular function, as well as the paramount role of cell damage in the early phase of AKI or even before it. The state-of-the-art in AKI gives multiple tools to complex assessment of actual kidney function, renal functional reserve, and stage of cellular damage in the context of clinical background. This combined analysis allows to grade the patients depending on the risk of AKI development, progression into serious AKI, CKD development, or recovery. However, serum creatinine remains the major index of AKI and the only biomarker put into everyday practice. Facing the future in prevention of pediatric AKI should mean identifying the patients at risk before the injury is fulfilled, preferably by assessing kidney functional reserve and using point-of-care testing before planned interventions. Coping with AKI diagnostics should concentrate on creation of sequential “AKI panels”, where various markers are evaluated according to the sequence of their appearance in serum/urine.

## Figures and Tables

**Table 1:** KDIGO acute kidney injury (AKI) and acute kidney disease (AKD) definitions [7,8]. eGFR, estimated glomerular filtration rate.

Stages of AKI	Serum Creatinine	eGFR	Urine Output (UOP)
Stage 1	1.5-fold increase of baseline creatinine in 7 days or increase by ≥0.3 mg/dL within 48 h	-	<0.5 mL/kg b.w./h in 6–12 h
Stage 2	2-fold increase	-	<0.5 mL/kg b.w./h in ≥12 h
Stage 3	3-fold increase or increase by ≥0.5 mg/dL within 48 h or serum creatinine ≥4.0 mg/dL	decrease to <35 mL/min/1.73 m^2^ in patients <18 years or receipt of renal replacement therapy	<0.3 mL/kg b.w./h in ≥24 h or anuria in ≥12 h
**AKD**	**Serum creatinine**	**eGFR**	**Markers of damage**
For ≤3 months	increase by >50%	decrease by ≥35%	present

**Table 2:** Stages of AKD according to Acute Disease Quality Initiative (ADQI) consensus [10].

Stages of AKD	Serum Creatinine	Markers of Damage
Stage 0A	Return to baseline values	No evidence of injury Risk of long-term events
Stage 0B	Return to baseline values	Ongoing kidney damage/injury Loss of renal reserve
Stage 0C	Increase less than 1.5-fold	Ongoing kidney damage/injury
Stage 1	1.5-fold increase	Ongoing kidney damage/injury
Stage 2	2-fold increase	Ongoing kidney damage/injury
Stage 3	3-fold increase	Ongoing kidney damage/injury
Ongoing RRT	Receipt of renal replacement therapy (RRT)	

## Data Availability

Not applicable.
